# Supplementary material for: Effects of Endochondral and Intramembranous Ossification Pathways on Bone Tissue Formation and Vascularization in Human Tissue-Engineered Grafts
Source: Cells. 2022 Sep 29;11(19):3070. doi: 10.3390/cells11193070 (PMC9564153; doi:10.3390/cells11193070)
Supplement: Supplementary file 1 [file cells-11-03070-s001.zip › cells-1905047-supplementary.pdf]

Effects of endochondral and intramembraneous ossification pathways on bone tissue formation and vascularization in human tissue-engineered grafts

SUPPLEMENTAL FIGURES

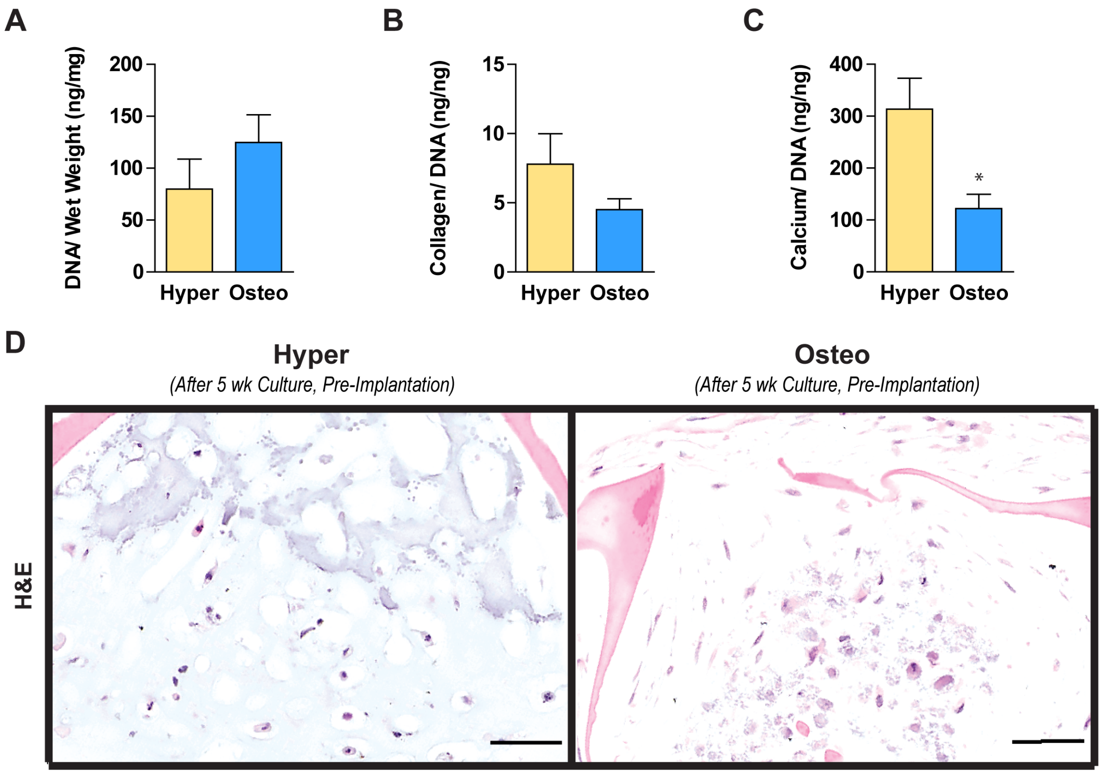

Figure S1: Construct composition and histomorphology after the 5 week culture (pre-implantation). A DNA content normalized to wet weight (n=4). **B** Collagen content normalized to the amount of DNA (n=4). **C** Calcium content normalized to the amount of DNA (n=3). **D** H&E staining demonstrating the construct morphology. Scale bars: 50 µm. Data are shown as average ± SD. * denotes significant differences between the groups ($p<0.05$).

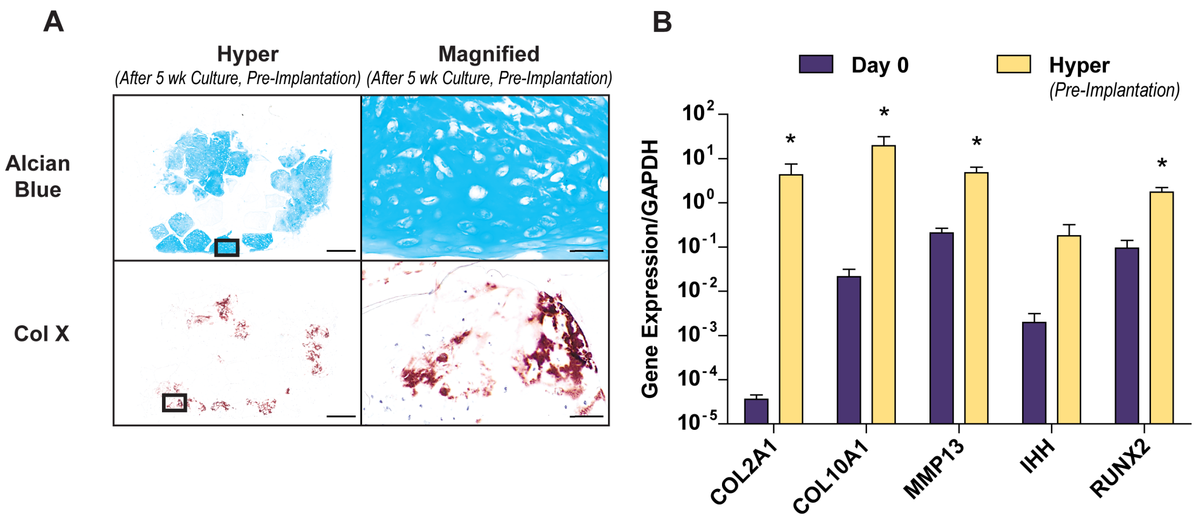

Figure S2: Validation of hypertrophic chondrocytes. **A** Histology and immunohistochemistry demonstrating the deposition of glycosaminoglycans (Alcian blue) and the hypertrophic chondrocyte specific type X collagen (Col X). Scale bars: 500 μm (top images) and 50 μm (magnified images). **B** Expression of key chondrogenesis and hypertrophy-related genes compared to the day 0 values (n=4). Data are shown as average ± SD. *Denotes significant differences between the groups (p<0.05).

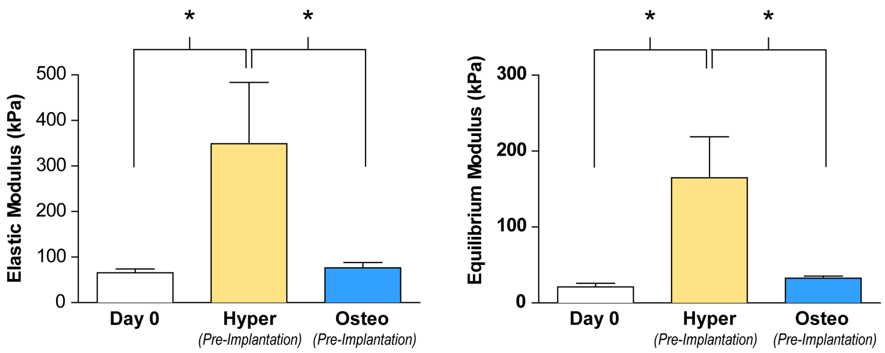

Figure S3: Construct mechanical properties pre-implantation. The elastic (Young's) and equilibrium moduli of each construct prior to cultivation (Day 0) and pre-implantation (Hyper, Osteo) (n=5). Data are shown as average ± SD. * denotes significant differences between the groups (p<0.05).

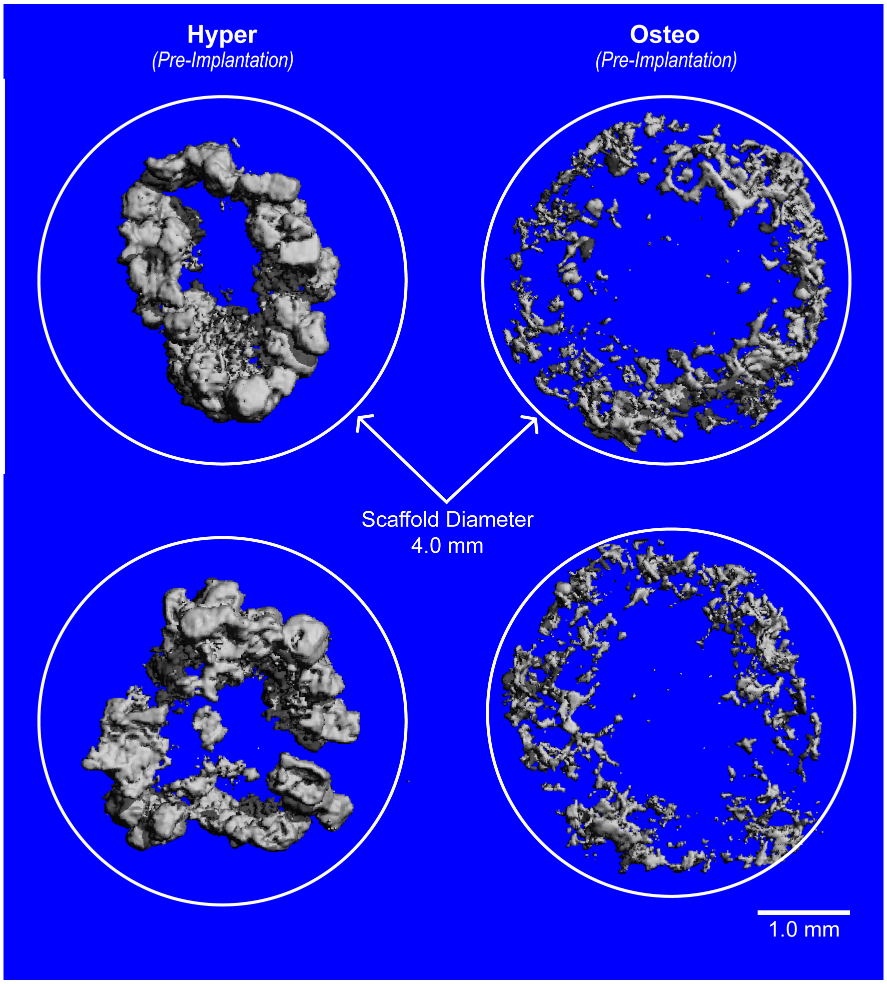

Figure S4: Mineral deposition in cultured constructs pre-implantation. μCT reconstruction of the pre-implantation constructs, with white circles denoting the size of the silk scaffold. Differences in the localization of the deposited mineral are noticeable, with hypertrophic-chondrocyte constructs displaying inner localization and the osteoblast constructs containing mineral closer to the periphery. Scale bars: 1.0 mm.

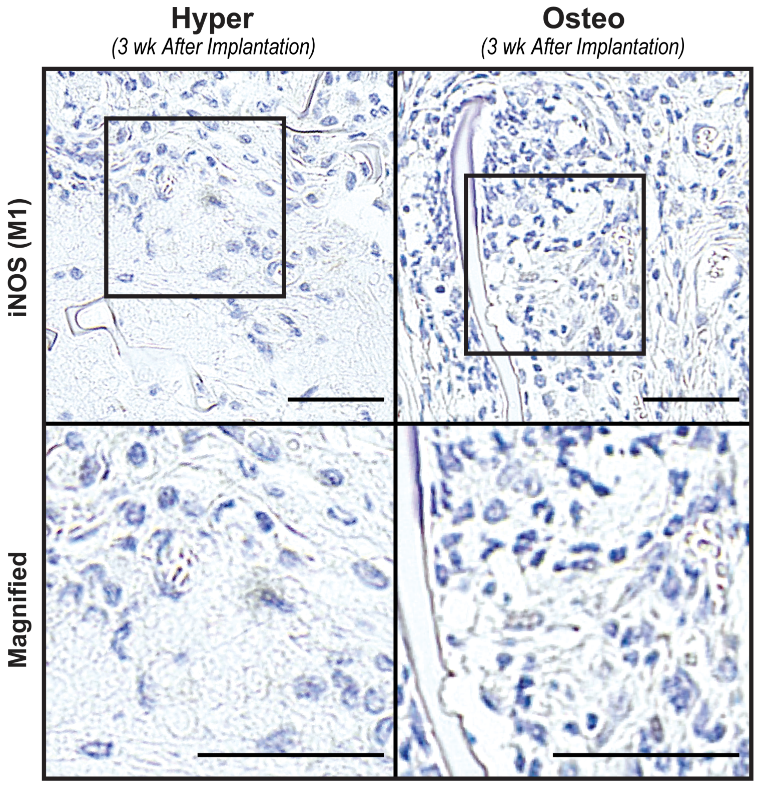

Figure S5: Presence of M1 macrophages. Both the hypertrophic chondrocyte and osteoblast constructs demonstrated minimal presence of M1 macrophages as visualized by iNOS immunohistochemistry harvested 3 weeks after implantation. Scale bars: 50 µm.

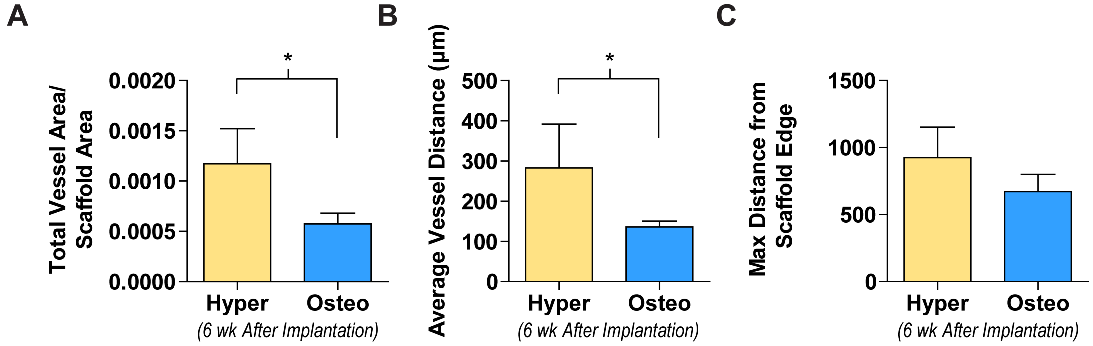

Figure S6: Vascularization. **A** Total vessel cross-sectional area normalized by the construct cross-sectional area. **B** The average vessel distance from the closest scaffold edge, demonstrating the infiltration of vessels into the interior of the construct. **C** The maximum distance recorded from a vessel to the nearest scaffold edge. Data are shown as average ± SD. * denotes significant differences between the groups ($p<0.05$).
